# The *Arabidopsis ANGUSTIFOLIA3*-*YODA* Gene Cascade Induces Anthocyanin Accumulation by Regulating Sucrose Levels

**DOI:** 10.3389/fpls.2016.01728

**Published:** 2016-11-22

**Authors:** Lai-Sheng Meng, Ying-Qiu Li, Meng-Qian Liu, Ji-Hong Jiang

**Affiliations:** ^1^The Key Laboratory of Biotechnology for Medicinal Plant of Jiangsu Province, School of Life Science, Jiangsu Normal UniversityXuzhou, China; ^2^Centre for Transformational Biotechnology of Medicinal and Food Plants, Jiangsu Normal University – Edinburgh UniversityXuzhou, China

**Keywords:** AN3/GIF1, *YODA* (*YDA*), sucrose levels, anthocyanin accumulations, *Arabidopsis*

## Abstract

Anthocyanin accumulation specifically depends on sucrose (Suc) signaling/levels. However, the gene cascades specifically involved in the Suc signaling/level-mediated anthocyanin biosynthetic pathway are still unknown. *Arabidopsis ANGUSTIFOLIA3* (*AN3*), a transcription coactivator, is involved in the regulation of leaf shape and drought tolerance. Recently, an *AN3-CONSTITUTIVE PHOTOMORPHOGENIC 1* gene cascade has been reported to regulate the light signaling-mediated anthocyanin accumulation. Target gene analysis indicates that *AN3* is associated with the *YODA* (*YDA*) promoter, a mitogen-activated protein kinase kinase kinase, *in vivo* for inducing anthocyanin accumulation. Indeed, loss-of-function mutants of *YDA* showed significantly increased anthocyanin accumulation. *YDA* mutation can also suppress the decrease in *an3-4* anthocyanin accumulation. Further analysis indicates that the mutations of *AN3* and *YDA* disrupt the normal Suc levels because of the changes of invertase activity in mutants of *an3* or *yda*, which in turn induces the alterations of anthocyanin accumulation in mutants of *an3* or *yda* via unknown regulatory mechanisms.

## Introduction

Anthocyanins are a class of flavonoids widely found in plants and are responsible for plant colouration (Winkel-Shirley, 2001; Gould et al., 2002). The antioxidant properties of anthocyanins can provide nutritional benefits for humans and can protect plants from oxidative damage (Nagata et al., 2003).

Anthocyanin biosynthesis has been diffusely investigated in plants (Gou et al., 2011; Meng, 2015). In many plants, for example, *Arabidopsis thaliana* (thale cress), *Antirrhinum majus* (snapdragon) and *Petunia hybrida* (petunia), early biosynthesis genes (EBGs), including *chalcone isomerase* (*CHI*), *chalcone synthase, flavonoid 3′-hydroxylase* (F3*′*H) and *flavanone 3-hydroxylase*, can be induced early compared with late biosynthesis genes (LBGs), including *leucoanthocyanidin oxygenase, dihydroflavonol 4-reductase, UDP-glucose*, and *anthocyanidin reductase* (Meng, 2015).

Anthocyanin biosynthesis has been widely affected by biotic and abiotic factors (Yokawa et al., 2014). Phytohormones, for example, gibberellins (Weiss et al., 1995), abscisic acid and auxin (Jeong et al., 2004; Hoth et al., 2010), jasmonates (Qi et al., 2011), cytokinin (Deikman and Hammer, 1995) and ethylene (Morgan and Drew, 1997; Jeong et al., 2010), modulate anthocyanin biosynthetic pathways in cell suspensions or/and whole plants differently (Ozeki and Komamine, 1986). Moreover, sugar (e.g., sucrose [Suc] and glucose) is regarded as transported carbohydrates and signal molecules in vascular plants (Lou et al., 2007). Along with the synergistic effect on mobilizing sugar usage and storage or sugar production, glucose signaling mediates carbohydrate availability and modulates metabolism (Baier et al., 2004). Anthocyanin biosynthesis is also significantly upregulated along with Suc concentration, and upregulation during anthocyanin biosynthesis specifically depends on Suc (Solfanelli et al., 2006; Jeong et al., 2010).

Although, a few reports have shown that anthocyanin accumulation is specific for Suc signaling, what gene cascade Suc is integrated to and how Suc is linked to the relative proteins are still unknown. A recent work (Meng, 2015) showed that *AN3* positively regulates anthocyanin accumulation. In this study, *yda* mutants exhibited higher anthocyanin accumulation than the wild-types. Molecular data indicated that *YDA* is a target gene of *AN3*. Moreover, *AN3* genetically acts upstream of *YDA* to regulate anthocyanin accumulation. Further analysis revealed that the mutations of *AN3* and *YDA* can disrupt the normal Suc levels as a result of higher or lower invertase (INV) activity in *an3* and *yda* mutants, respectively. Thus, lower and higher anthocyanin accumulations were observed in *an3* and *yda* mutants, respectively.

## Materials and Methods

### Plant Materials and Growth Conditions

*an3-1, an3-4* (Horiguchi et al., 2005), *yda-1, emb71*, and *yda-2* (Lukowitz et al., 2004; Kang et al., 2009; Meinke et al., 2009) mutants, *35S:AN3:3XGFP* (Kanei et al., 2012) transgenic plants with Col-0 background were described previously. *emb71* is an allele of *yda* (*yda1* and *yda2*).

The *an3-1* (AT5G28640, CS241), *yda-1* (AT1G63700, CS6392), *yda-2* (AT1G63700, CS6393), *emb71* (AT1G63700, CS84618), and *grf1* (AT2G22840, SALK_069339C) were obtained from the ABRC (Ohio State University). Professor G. Horiguchi (Rikkyo University, Japan) kindly provided via the *35S:AN3:3XGFP* seeds. Prof. H. G. Nam (DGSIT, Korea) kindly provided the *an3-4* mutant seeds. Prof. H. Q. Yang (ShangHai JiaoTong University, China) kindly provided the *pHB-YDA:GFP* plasmid.

The *an3 yda* double mutant was gained by *an3-4* and *yda-1* phenotype. The above method about the double mutant construction was described previously (Meng and Yao, 2015). By using the *Agrobacterium tumefaciens*-mediated floral dip method, we gained transgenic plants (Meng and Liu, 2015; Meng et al., 2015a).

*ProYDA:GUS*/*an3-4* lines were obtained, as was described by Meng and Yao (2015). We introduced *pMD111-Pro YDA:GFP* (*YDA pro:GFP* with two TCTCTC motif or *YDA pro:GFP* without two TCTCTC motif) into the *35S:AN3* background, as were described previously (Meng and Yao, 2015). Transformants were selected on Hygromycin B for three generations and analysis of segregation ratios.

Condition of *Arabidopsis* plant growth was described by Meng and Yao (2015) and Meng L. et al. (2016).

### Observation of Root System

*Arabidopsis* roots were photographed under relevant magnification using a HIROX three-dimensional video microscope (Meng L.S. et al., 2016).

### Constructing Plasmid

For *AN3* and *YDA* promoter analysis, we constructed relative plasmid, as was described by Meng and Yao (2015). Obtaining *35S:AN3-HA* plasmid was described by Meng and Yao (2015). Obtaining *pGWB5 /AGL103-YDA-GFP* plasmid and obtaining *pMD111-PAP1-GFP* plasmid (containing TTCAAA motif) was described by Meng and Yao (2015).

### GUS Expression

GUS assay was described by Meng et al. (2015a,b).

### Confocal Laser Scanning Microscope for GFP Imaging

Confocal laser scanning microscope for GFP Imaging was described by Meng (2015).

### ChIP Assay

ChIP assay was described by Meng (2015). The ChIP DNA products were analyzed through qPCR via using five pairs of primers that were synthesized to amplify ∼300-bp DNA fragments of the promoter and CDS region of *YDA* in ChIP analysis. The primer sequences of *YDA* were P1-tgt gtc act aac tca ctt cac; P2-gaa aac cct aag tag aac aac; P3-gct ttc gat ttg att cca ttt caa; P4- tca atg tga tct tca acc ta; P5- tac aaa gat taa cgc acc aaa gg; and P6-tca aaa gca atc gaa gaa tcc aa; P7-AAT CAT TAT AAA AGT CAG CAA CTA A; P8-AAG AAT ATG AAA TAC TTC CAA TTC A. The primer sequences were 5′-GCA GCA AGA TCG GTC GCG GA-3′ and 5′-ACC GCC ACC ACC ACT TCC CA-3′ for CDS region of *YDA*. The primer sequences of *F3*′*H (TT6)* were F1-(F1-ttg taa aca aca acg aaa act gaa; R1-aac ggc aac ggc tca tct tc); F2 (F2-cgc aag ccc gta cca gaa cat g; R2-tac agt ttc gtt tac acc gta g). The primer sequences of *FLS* were F1 (F1-ctc tct ctc tct cgc tct ca; R1-aac acc tgt ata aac aaa aaa gg); F2 (F2-tta tgg cct aaa ata taa taa cat c; R2-ata tgt gta agt cgt cga ggc). The primer sequences of *CHI* were C1 (F1-ctt gat tct tga tat tta tac cgc; R1-ttg tca gac caa ttc taa tcc ga); C2 (F2-caa ttg gcg act act ttc agt aa; R2-tta cac gta aca cgt tgg gaa at). The primer sequences of *UBQ5* were F1—gac gct tca tct cgt cc; R1-gta aac gta ggt gag tcc a).

### Real-Time Polymerase Chain Reaction (PCR)

Total RNA was extracted and real-time polymerase chain reaction (PCR) was performed, as described by Meng et al. (2015). For analyzing *EMB71*/*YDA* expression level in 2-week-old Col-0, *an3-1, an3-4*, and *35S:AN3*/*an3-4* seedlings, primers F-5′-GCA GCA AGA TCG GTC GCG GA-3′ and R-5′-ACC GGG TCT CAG GTC GAG GG-3′ were used. For analyzing *AN3* expression level in 2-week-old Col-0, *emb71*, L*er, yda-1*, and *yda-2* seedlings, primers F-5′-GCC TCA GCC ACC AAG TGT GCA T-3′ and R-5′-ACC GCC ACC ACC ACT TCC CA-3′ were used. For analyzing *FLS, CHI, F3’H* (*TT6*), *TT19, UGT79B1*, and *UGT89B1* expression levels in 2-week-old Col-0, *an3-4*, and *emb71* seedlings, primers F-5′-ATG GAG GTC GAA AGA GTC CAA-3′ and R-5′-TTT TCT TCC GAC GTC TTG TAA AC-3′ were used for *FLS*; primers F-5′-ATG GCT AAA CCC ACA TCA CGA-3′ and R-5′-GGT TTA GTA TAG AGT TAA AGA AGT-3′ were used for *CHI*; primers F-5′-ATG GCT CCA GGA ACT TTG ACT-3′ and R-5′-CAC GAG CGA GAC GAG TCA TAT-3′ were used for *F3*′*H* (*TT6*); primers F-5′-ATG GTT GTG AAA CTA TAT GGA CA-3′ and R-5′-CTA GAG ACT TGC CCA AAA GGT-3′ were used for *TT19*; primers F-5′-ATG GGT GTT TTT GGA TCG AAT G-3′ and R-5′-GAA GAA AGG GAC GTC GGA GT-3′were used for *UGT79B1*; and primers F-5′-ATG ACA ACA ACA ACA ACG AAG AA-3′ and R-5′-TAT AGC TTC GAG AGG AAG TTG CT-3′ were used for *UGT89B1.* The primer sequences of *UBQ5* were F1-gac gct tca tct cgt cc; R1-gta aac gta ggt gag tcc a. *UBQ5*, a stable internal control gene, has been proven (Jain et al., 2006). The primer sequences of *UBQ5* were F1—gac gct tca tct cgt cc; R1-gta aac gta ggt gag tcc a. *UBQ5*, a stable internal control gene, which has been proved by using the methods of Vandesompele et al. (2002).

### Test of Sugar Metabolites

Twelve-day-old seedlings were ground in liquid nitrogen. Then, the powder was dissolved with 1 mL of 80% ethanol for 1 h. These extracts were centrifuged at 12,000 × *g* for 10–15 min. The supernatant and ethanol buffer were transferred to a fresh tube and evaporated under vacuum to dry for 40–60 min, and the residues were redissolved in 600 μL of ddH_2_O and kept at 70°C for 10–15 min.

Using chloroform/isoamyl alcohol (24:1, *v*/*v*), the aqueous fraction was extracted two to three times before high-performance liquid chromatography (HPLC) analysis. Sugars were quantified and identified via chromatography on an Agilent carbohydrate column and tested with a refractive index detector (Altex 156, Altex Scientific, Inc., Berkeley, CA, USA). Concentrations were measured from the integrated peak area and the peak heights using Suc, fructose and glucose (20 mg/mL) as standard samples (Kovtun et al., 2000).

### Anthocyanin Measurement

Anthocyanin extraction and measurement have been described previously by Meng (2015).

### Neutral INV Assay via Enzyme-Linked Immunosorbent Assay (ELISA)

Neutral INV in *Arabidopsis* plants was extracted and purified according to the method described previously by Lou et al. (2007). Enzyme-linked immunosorbent assay (ELISA) was used to assay INV activity. Briefly, the plant neutral INV activity assay kit (Sangon, Shanghai, China) was used. According to the manufacturer’s protocols, in assay medium (48-well plates) with neutral INV antigen, normal and assayed samples (antibody) were added and incubated at 37°C for 30 min. Then, the mixture was washed five times using a cleaning solution, and HRP (INV label) was applied and incubated at 37°C for 30 min to produce antigen–antibody–HRP complexes. After the reaction, the mixture was washed five times using a cleaning solution, and TMB-A and TMB-B were added and incubated at 37°C for 10 min for staining. TMB was catalyzed by HRP, and the mixture turned blue. When a termination buffer was applied, the mixture turned yellow. An enzyme mark instrument was used to assay the optical density values (450 nm). Finally, relative to a normal sample, the neutral INV activity of the assayed samples was estimated.

### INV Activity Localisation

For localizing INV activity on *Arabidopsis* plants, histochemical staining method is performed based on a few coupled redox reactions to produce a non-soluble blue formazan precipitate, as described by Kuhn (1999) with minor modification. Five-day-old *Arabidopsis* seedlings were fixed in 4% formalin (pH 7.0–7.5) for 1 h. Then, fixed seedlings were rinsed several times in a few alterations of water and overnight to remove all endogenous sugars. After this rinsing process, these rinsed materials were incubated in a reaction mix buffer with 0.38 M disodium hydrogen phosphate buffer (pH 6.0), 15 units of glucose oxidase (Transgene SA, Illkirch, Strasbourg, France), 0.015% (wt/vol) phenazine methosulfate, 0.030% (wt/vol) nitroblue tetrazolium (NBT) and 1% Suc at 37°C under darkness. In the control reactions, Suc was not added. Reaction termination after 2–3 h was performed by water rinsing and post-fixing in 4% formalin for 30 min.

## Results

### Seedlings and Seeds of *yda* Mutants Showed Higher Anthocyanin Accumulation than the Wild-Types

**FIGURE 1:**
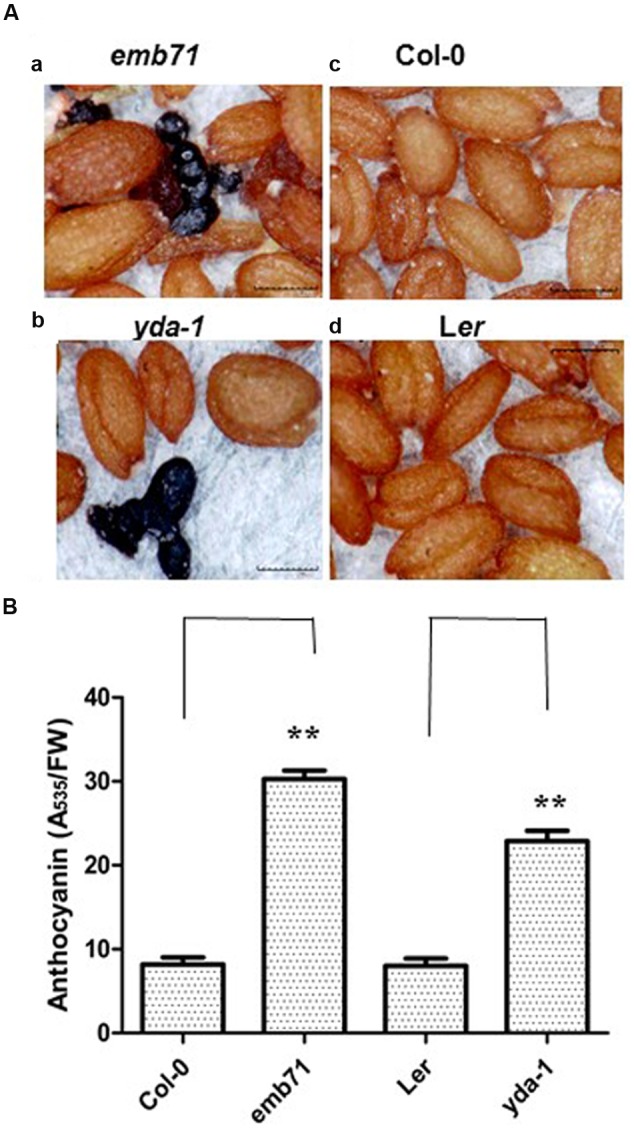
***yda* seeds and seedlings had increased anthocyanin accumulation. (A)** Representative mature dry seeds *emb71* (a), *yda-1* (b), Wild-type (Col-0) (c), and L*er* (d). Materials were from at least five independently propagated lines. Magnifications are the same. **(B)** Bar graph exhibiting the difference in the anthocyanin accumulation between the 12-day-old *emb71, yda-1*, Wild-type (Col-0) and L*er* seedlings grown under white light conditions. Anthocyanin content was assayed via taking the absorbance of plant extracts at 535 nm over grams of fresh weight (FW). Error bars represent SD (*n* = 8). Heteroscedastic *t*-test analysis showed significant differences (^∗∗^*P* < 0.01). This experiment was repeated at least three times with similar results (biological replicates).

Recently, *Arabidopsis AN3* has been reported to be a positive regulator of anthocyanin accumulation (Meng, 2015), and *Arabidopsis AN3-YDA* has been reported to form the gene cascade for the regulation of drought tolerance (Meng and Yao, 2015). Therefore, whether *YDA* is also involved in the regulation of anthocyanin accumulation should be determined. Indeed, heterozygous mature seeds for the *emb71* mutant (its stock number at the *Arabidopsis* Biological Resource Center is CS84618, and its gene locus is AT1G63700) can generate phenotypical segregation and form homozygous seeds with dark purple colouration (approximately 24.2%; **Figure 1Aa,c**) (Meinke et al., 2009). *emb71* is an allele of *yda* (*yda1* and *yda2*). Heterozygous mature seeds for the *yda-1*/*+* (CS6392) mutant, a nonsense mutation truncating the protein within the catalytic domain (Lukowitz et al., 2004), were observed to determine whether alleles of *YDA* behave similarly. Indeed, *yda-1*/*+* seeds can also generate phenotypical segregation and form homozygous seeds with dark purple colouration (<5%; **Figure 1Ab,d**). The anthocyanin concentration in *yda* seedlings was determined to confirm this finding. Subsequent findings indicated that the *yda* seedlings exhibited a higher anthocyanin content than the wild-type seedlings (**Figure 1B**). The 7-day-old wild-type seedlings were purplish at the primary roots, and the corresponding *emb71* mutant roots exhibited excess anthocyanin accumulation (Supplementary Figure S1). Taken together, these findings revealed that *YDA* may negatively regulate anthocyanin accumulation.

### *YDA* Is a Target Gene of *AN3* in the Regulation of Anthocyanin Accumulation

**FIGURE 2:**
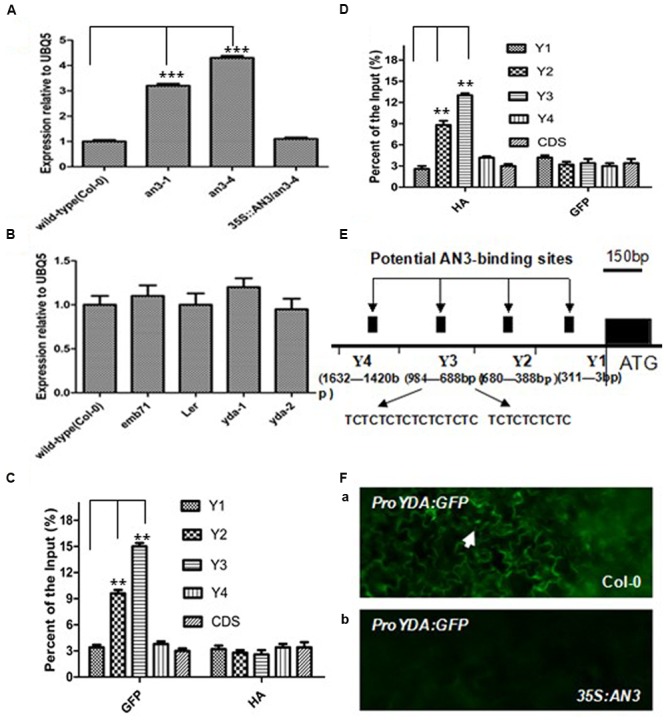
**AN3 negatively regulates *EMB71/YDA* expression. (A)** Bar graph exhibiting the difference of expression of *EMB71/YDA* between at least 10 Wild-type (Col-0), *an3-1, an3-4*, and *35S:AN3/an3-4* seedlings grown under white light conditions. **(B)** Bar graph exhibiting the difference of expression of AN3 between Wild-type (Col-0), *emb71*, L*er, yda-1*, and *yda-2* seedlings grown under white light conditions; Data were from quantitative RT-PCR. Materials were from seedlings of at least five independently propagated lines. And wild-type is set as 1.0. Quantifications were normalized to the expression of *UBQ5*. Error bars represent SD (*n* = 3). Heteroscedastic *t*-test analysis showed significant differences (^∗∗∗^*P* < 0.001). In **(A,B)** these experiments were repeated at least two times with similar results (technical replicates). **(C)** A chromatin immunoprecipitation (ChIP) analysis. Enrichment of particular *YDA* chromatin regions with anti-HA antibody (as a control) or anti-GFP antibody in *35S:AN3-3XGFP* transgenic plants as detected by real-time PCR analysis. **(D)** A chromatin immunoprecipitation (ChIP) analysis. Enrichment of particular *YDA* chromatin regions with anti-GFP antibody (as a control) or anti-HA antibody in *35S:AN3-HA* transgenic plants as detected by real-time PCR analysis. Quantifications were normalized to the expression of *UBQ5*. Error bars represent SD (*n* = 3). Heteroscedastic *t*-test analysis showed significant differences (^∗∗^*P* < 0.01). Input is set as 100% [supernatant including chromatin (input material) is considered as 100%, immunoprecipitated chromatin/input material X 100% for enrichment product of particular *YDA* chromatin regions]. In **(C,D)** these experiments were repeated at least two times with similar results (biological replicates). **(E)** Schematic of the *YDA* promoter loci and their amplicons for ChIP analysis. **(F)** *ProYDA:GFP* in WT (Col-0) (a) and *35S:AN3* (b) cotyledons. they are at same magnification. White arrows point to GFP-positive nuclei.

Pertinent experiments were performed to determine whether *YDA* was directly modulated via *AN3* at the transcript level for inducing anthocyanin accumulation. As speculated, the transcript level of *YDA* was higher in *an3-4* seedlings than in wild-type seedlings (**Figure 2A**). Meanwhile, *AN3* transcript accumulation among the Col-0, *emb71, yda-1*, L*er*, and *yda-2* seedlings was analyzed. The observations indicated that the *AN3* transcript level in wild-type seedlings was comparable with that in mutant seedlings (**Figure 2B**). Thus, *AN3* might negatively regulate the *YDA* expression at the transcript level.

**FIGURE 3:**
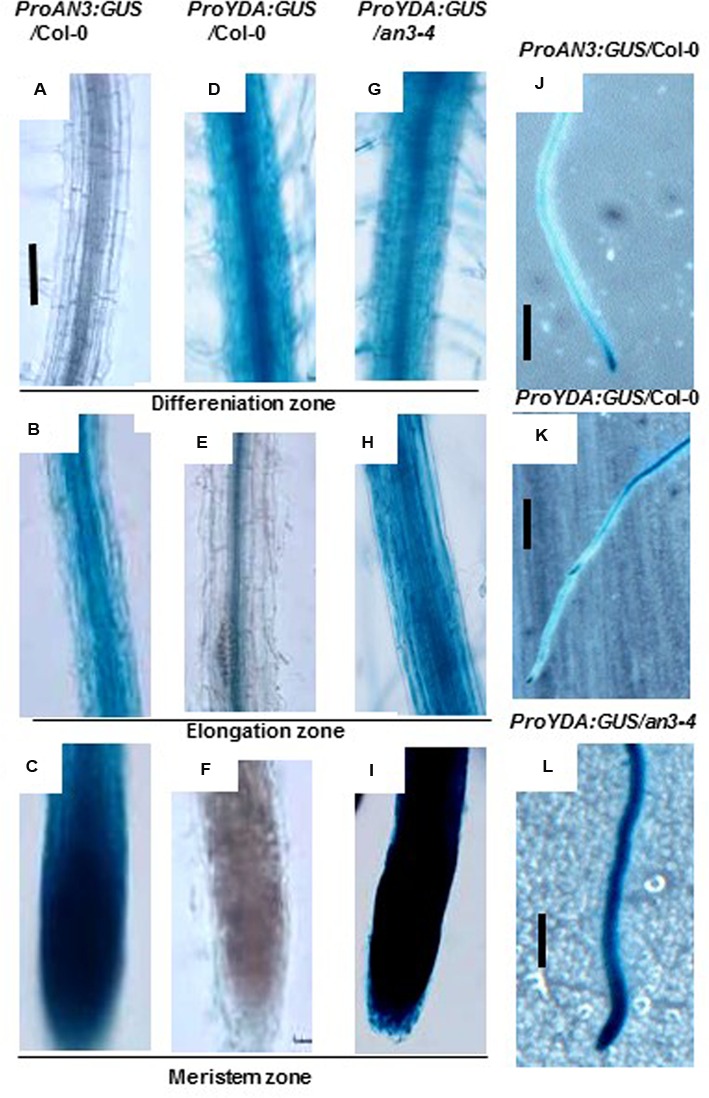
**AN3 negatively regulates *EMB71/YDA* expression. (A–C)** Representative ProAN3:GUS expression on root differentiation, elongation, and meristem zone in the wild-type background, respectively. **(D–F)** Representative *ProEMB71/YDA:GUS* expression on root differentiation, elongation, and meristem zone in the wild-type background, respectively. **(G–I)** Representative *ProEMB71/YDA:GUS* expression on root differentiation, elongation, and meristem zone in the *an3-4* background, respectively. Bar = 100 um. **(J–L)** Representative *ProAN3:GUS, ProEMB71/YDA:GUS* and *ProEMB71/YDA:GUS* expression on primary roots in the wild-type background, in the wild-type background and in the *an3-4* background, respectively. Bar = 2.0 mm. Transgenic plants with GUS were subjected to GUS staining for 8 h.

The *proAN3-GUS* expression levels were evidently observed in the meristem region of the Col-0 roots, but were only weakly detected in the differentiation and elongation regions of the Col-0 roots (**Figures 3A–C,J**). These findings are consistent with those of previous reports (Horiguchi et al., 2005, 2011), that is, the *AN3* expressions were more evidently observed in young organs (e.g., young leaf meristem) than in mature organs (e.g., mature leaf blade). Meanwhile, the *proYDA-GUS* expression levels were significantly observed in the differentiation and elongation regions of the Col-0 roots, but were only weakly detected in the meristem region of the Col-0 roots (**Figures 3D–F,K**). By contrast, the *proYDA-GUS* expression levels can be significantly detected in all sections of the *an3-4* roots (**Figures 3G–I,L**). These results confirmed that *AN3* negatively regulates *YDA* at the transcriptional level.

**Figure 2E** shows that only a few amplicons exist in the *YDA* promoter, which is used for chromatin immunoprecipitation (ChIP) analyses. These amplicons have two TCTC motifs (TCTCTCTCTCTCTCTC and TCTCTCTCTC), which span from -850 to -950 (**Figure 2E**) (Meng, 2015; Meng and Yao, 2015). ChIP-PCR analysis was performed to determine whether *AN3* is associated with the *YDA* promoter of these TCTC motifs. Transgenic seedlings with the *35S:AN3-3XGFP* and *35S:AN3-HA* constructs were used. Amplicons of Y3 containing two motifs (Meng and Yao, 2015) (TCTCTCTCTC and TCTCTCTCTCTCTCTC), which span from -883 to -893 and from -910 to -934 in the *YDA* promoter, were highly enriched using the anti-GFP and anti-HA antibodies (**Figures 2C,D**). The amplicon of Y2, which spans from -388 to -680 in the *YDA* promoter, was adjacent to, but did not contain, two TCTC motifs, and the amplicons of Y1 and Y4, which span from -3 to -311 and from -1,420 to -1,632 in the *YDA* promoter, respectively, were distal to two TCTC motifs (**Figure 2E**). Consequently, region Y3 primers resulted in the largest amount of PCR product, and the amount of PCR amplification product was reduced with primers for region Y2 and even more so with primers for regions Y1, Y4 and CDS (**Figures 2C,D**). These findings indicate that *AN3* is associated with the *YDA* promoter *in vivo*, which is necessary for *AN3* to suppress *YDA* expression. In addition, *AN3* cannot bind directly to *YDA* promoter sequences in a gel mobility shift assay *in vitro* (unpublished data). At 10 days post-germination, wild-type plants (Col-0) can express *YDA pro:GFP* containing TCTC motifs (10 out of 12), but overexpressing lines (*35S:AN3*) cannot (0 out of 12; **Figure 2Fa,b**). Taken together, these findings showed that the TCTC motifs in the *YDA* promoter are essential for *AN3* to suppress the *YDA* promoter. Indeed, *AN3* is associated with the *YDA* promoter *in vivo*.

### *an3* and *yda* Are Mutants of Sugar Metabolism and/or Sugar Signaling

In determining the type of signaling/metabolism that mediates the *AN3-YDA* gene cascade pathway, *an3* and *yda*, as signaling/metabolism mutants, should be appropriately classified. Microarray data (Horiguchi et al., 2011) showed many sugar-related genes with at least twofold higher expression levels in the *an3-4* seedlings than in the wild-type seedlings. Horiguchi et al. (2011) reported that the altered gene expression in *an3-4* plants mostly influenced metabolism, not developmental regulation. Lukowitz et al. (2004) conducted a microarray analysis of *yda* mutants and indicated that only 14 of 8,000 genes exhibited changes in expression levels of at least twofold, and these genes included those involved in sugar-mediated signaling pathways (AT3G27660), sugar metabolism (AT5G57550, AT2G43570, and AT4G15760), Suc response (AT5G13930) and cell wall synthesis (At2g45220). That is, more than half are closely related to sugar metabolism and/or sugar signaling. In other microarrays of *yda* mutants (Bergmann et al., 2004), genes encoding the cell wall of differentiated epidermis were extensively reinforced. For example, more than 11% of the genes upregulated in *yda* mutants are involved in cell wall differentiation. These results indicated that *AN3* and *YDA* might be involved in sugar metabolism and/or sugar signaling.

**FIGURE 4:**
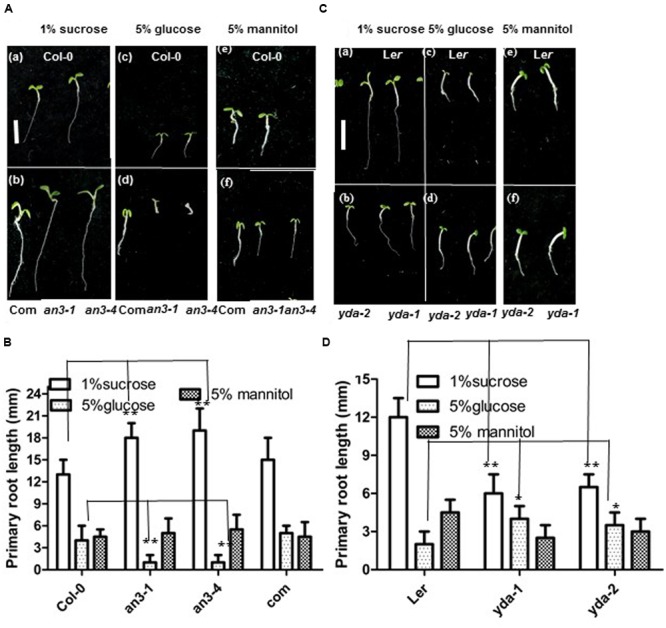
**AN3 and EMB71/YDA are involved in sugar metabolism and/or sugar signaling. (A)** Representative 5-day-old Wild-type (Col-0) (a), complementary line (*35S:AN3/an3-4*) (b), *an3-1* (b) and *an3-4* (b) seedlings grown on solid MS medium with 1% sucrose; and 5-day-old Wild-type (Col-0) (c), complementary line (*35S:AN3/an3-4*) (d), *an3-1* (d) and *an3-4* (d) seedlings grown on solid MS medium with 5% glucose; and 5-day-old Wild-type (Col-0) (e), complementary line (*35S:AN3/an3-4*) (f), *an3-1* (f), and *an3-4* (f) seedlings grown on solid MS medium with 5% mannitol. Bar = 5.0 mm. Com, complementary line. **(B)** Bar graph exhibiting the difference in the root length between Wild-type (Col-0), *an3-1, an3-4* and complementary line (*35S:AN3/an3-4*) seedlings grown on solid MS medium with 1% sucrose, 5%glucose, and 5% mannitol, respectively. Error bars represent SD (*n* = 20). Heteroscedastic *t*-test analysis showed significant differences (^∗∗^*P* < 0.01). Experiments were repeated three times with similar results (biological replicates). **(C)** Representative 5-day-old L*er* (a) and *yda-1, yda-2* (b) seedlings grown on solid MS medium with 1% sucrose; and 5-day-old L*er* (c) and *yda-1, yda-2* (d) seedlings grown on solid MS medium with 5% glucose; and 5-day-old L*er* (e) and *yda-1, yda-2* (f) seedlings grown on solid MS medium with 5% mannitol. Bar = 5.0 mm. **(D)** Bar graph exhibiting the difference in the root length between L*er, yda-1*, and *yda-2* seedlings grown on solid MS medium with 1% sucrose, 5% glucose, and 5% mannitol, respectively. Error bars represent SD (*n* = 20). Heteroscedastic *t*-test analysis showed significant differences (^∗∗^*P* < 0.01; ^∗^*P* < 0.05). Experiments were repeated three times with similar results (biological replicates).

**FIGURE 5:**
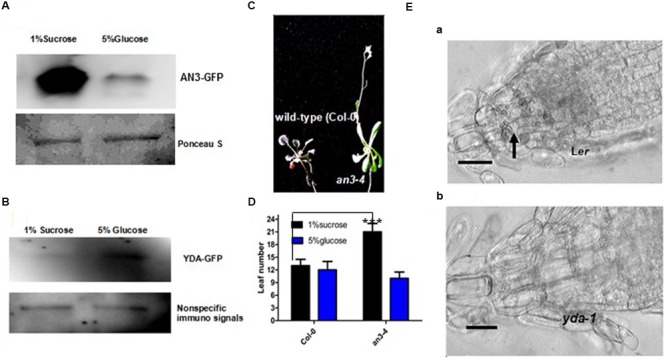
**AN3 and EMB71/YDA are Involved in Sugar Metabolism and/or Sugar Signaling. (A)** Representative 5% glucose treatment accelerates AN3 protein degradation. The 12-day-old *35S:AN3:3X:GFP* seedlings grown on solid MS medium with 1% sucrose and 5% glucose. Materials were from at least 10 independently propagated lines. **(B)** Representative 5% glucose treatment stabilizes EMB71/YDA protein. The 12-day-old *PHB:EMB71/YDA:GFP* seedlings grown on solid MS medium with 1% sucrose and 5% glucose. Materials were from at least 10 independently propagated lines. **(C)** Representative 5% glucose can restore the *an3-4* delayed flowering. Materials were grown on MS medium with 5% glucose for 4 weeks under long light (16 h light/8 h dark). Seedlings were from the same plate. Magnifications are the same. **(D)** Bar graph exhibiting the difference in the rosette leaf number between Wild-type (Col-0) and *an3-4* seedlings grown on solid MS medium with 1% sucrose and 5% glucose for 4 weeks under long light (16 h light/8 h dark). Error bars represent SD (*n* = 12). Heteroscedastic *t*-test analysis showed significant differences (^∗∗∗^*P* < 0.001). Experiments were repeated two times with similar results (biological replicates). **(E)** Longitudinal sections of roots of 8-day-old L*er* (a) and *yda-1* (b) seedlings. Seedlings were from the same plate. Magnifications are the same. Results are typical of those for many seedling. The arrow indicates starch grains.

The growth responses of *an3* and *yda* roots to high concentrations of glucose were assayed to validate this speculation. When grown on MS medium with 1% Suc, the *an3* seedlings exhibited an elongated root length compared with the wild-type and complementary lines (*35S:AN3*/*an3-4*) (**Figures 4Aa,b,B**). However, when grown on MS medium with 5% glucose, but not 5% mannitol, the *an3* seedlings exhibited a shortened root length compared with the wild-type and complementary lines (*35S:AN3*/*an3-4*), indicating that the response of *an3* seedlings to high concentrations of glucose was hypersensitive and was metabolic, rather than osmotic, stress (**Figures 4Ac–f,B**). In addition to glucose sensing, the protein levels of AN3-3XGFP in the *35S:AN3-3XGFP* seedlings were lower on solid MS medium with 5% glucose than with 1% Suc (**Figure 5A**), indicating that a higher concentration of glucose enhances *AN3* degradation. Moreover, the *an3* mutants have more rosette leaves than the wild-types at bolting [*an3-4* mutant = 21.3 ± 2.2, wild-type (Col-0) = 13.3 ± 1.5 (22); ^∗∗∗^*P* < 0.001] (Horiguchi et al., 2005), implying that *an3* mutants exhibit delayed flowering. Given that the flowering defect indicates the ability of the mutant to counteract the influence of sugars on flowering time (Zhou et al., 1998), whether the enhanced number of rosette leaves in *an3* mutant plants can be restored by 5% glucose should be determined. As speculated, the *an3* mutant plants had a similar number of rosette leaves to the wild-type plants on solid MS medium with 5% glucose (**Figures 5C,D**), indicating that the increase in *an3* rosette leaf number is dependent on the glucose signal. This phenotype is opposite to *glucose insensitive 1*, a glucose-insensitive mutant, indicating that flowering time is not delayed by glucose signaling/metabolism (Zhou et al., 1998). In contrast to the *an3* mutants, the *yda* seedlings showed dramatically shortened root length than the L*er* seedlings when grown on solid MS medium with 1% Suc (**Figures 4Ca,b,D**) (Lukowitz et al., 2004). However, when grown on solid MS medium with 5% glucose, but not 5% mannitol, *yda* plants exhibited an elongated root length compared with L*er* plants, implying that the response of *yda* seedlings to high concentrations of glucose was less sensitive and was metabolic, rather than osmotic, stress (**Figures 4Cc–f,D**). In addition to glucose sensing, the protein levels of YDA-GFP in the *PHB:YDA-GFP* seedlings were higher on solid MS with 5% glucose than that with 1% Suc (**Figure 5B**; Supplementary Figure S2). Notably, the root phenotype of the *yda-1* mutant is similar to general carbon starvation brought about by the reduced capacity for Suc catabolism in root cells, such as the loss of starch from the root cap (**Figure 5Ea,b**), and the extreme reduction in root growth (**Figure 4C**; Lukowitz et al., 2004), which is due to the suppression of cell elongation in the root elongation zone [L*er* = 360 ± 66 μm (*n* = 82); *yda-1* = 220 ± 43 μm (*n* = 78)].

### *an3* and *yda* Are Not Other Plant Hormone Signaling/Metabolism Mutants

Given that plant hormones, such as auxin and abscisic acid (Jeong et al., 2004; Hoth et al., 2010), cytokinin (Deikman and Hammer, 1995) and gibberellins (Weiss et al., 1995), differentially regulate anthocyanin biosynthesis in whole plants and in cell suspensions (Ozeki and Komamine, 1986), whether *an3* and *yda* are the signaling mutants of these plant hormones should be determined. The responses of *an3-1* roots to exogenously applied hormones were assessed, and the responses of the assayed hormones (i.e., ABA, GA, IAA, NAA, KT, and 6-BA) did not significantly restore the growth defects of *an3-1* roots (Supplementary Figure S3). Similarly, general hormone signaling did not restore the growth defect of *yda* roots, as proposed in a previous study (Lukowitz et al., 2004). Taken together, these findings indicated that *an3* and *yda* are sugar signaling/metabolism mutants, but not other plant hormone signaling mutants.

### The Decreased or Increased Anthocyanin Accumulation in the *an3* or *yda* Mutants Is a Result of the Decreased or Increased Endogenous Suc Accumulation, Respectively

**FIGURE 6:**
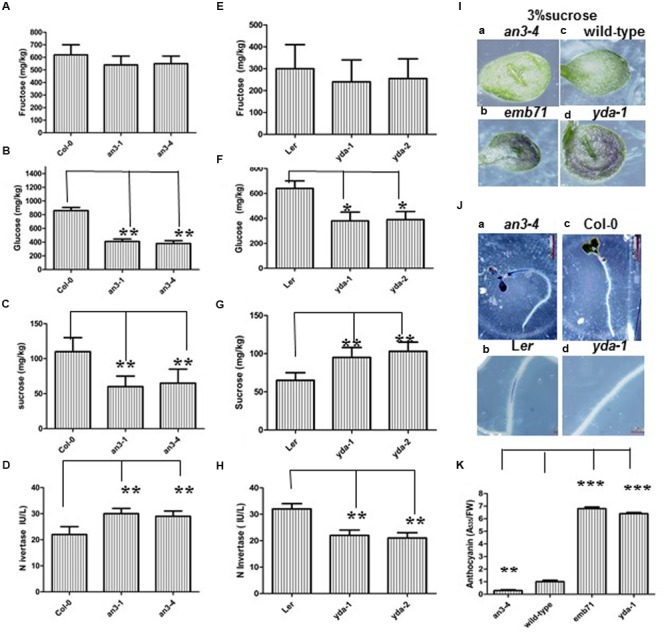
**Abnormal Anthocyanin Accumulation in *an3* and *yda* Mutants is Caused by Abnormal Endogenous Sucrose Accumulation. (A–C)** Bar graph exhibiting the difference of concentrations of hexose (glucose and fructose) **(A,B)** and sucrose **(C)** between Wild-type (Col-0), *an3-1* and *an3-4* seedlings. **(D)** Bar graph exhibiting the difference of the invertase activity between Wild-type (Col-0), *an3-1* and *an3-4* seedlings. IU/L indicates that seedling mixture per liter can produce the number of the invertase activity. **(E–G)** Bar graph exhibiting the difference of concentrations of hexose (glucose and fructose) **(E,F)** and sucrose **(G)** between L*er, yda-1* and *yda-2* seedlings. **(H)** Bar graph exhibiting the difference of the invertase activity between L*er, yda-1* and *yda-2* seedlings. IU/L indicates that seedling mixture per liter can produce the number of the invertase activity. The above seedlings were grown under long light (16L/8D) conditions on MS medium supplemented with 1% sucrose, respectively. Error bars represent SD (*n* = 3). Heteroscedastic *t*-test analysis showed significant differences (^∗∗^*P* < 0.01; ^∗^*P* < 0.05). Experiments were repeated two times with similar results (biological replicates). **(I)** Representative cotyledons of the 8-day-old *an3-4* (a), *emb71* (b), wild-type (c), and *yda-1* (d) seedlings grown under long light (16L/8D) conditions on MS medium supplemented with 3% sucrose. **(J)** Representative nitroblue tetrazolium (NBT) precipitation in the 6-day-old *an3-4* (a), L*er* (b), Col-0 (c), and *yda-1* (d) roots grown under long light (16L/8D) conditions on MS medium supplemented with 1% sucrose. Magnifications are the same. **(K)** Bar graph exhibiting the difference in the anthocyanin accumulation between the *an3-4*, Wild-type (Col-0), *emb71* and *yda-1* cotyledons grown under white light conditions on MS medium supplemented with 3% sucrose. Total amount of anthocyanin content in per gram fresh weight of Col-0 seedlings is 8.2. Wild-type (Col-0) is set as 1.0. Error bars represent SD (*n* = 3). Heteroscedastic *t*-test analysis showed significant differences (^∗∗∗^*P* < 0.001; ^∗∗^*P* < 0.01). Experiments were repeated two times with similar results (technical replicates).

Given that sugar is an important regulator of the expression of genes encoding metabolic enzymes and proteins involved in anthocyanin biosynthesis (Baier et al., 2004), and the results indicate that *an3* and *yda* are sugar signaling/metabolism mutants, whether lower or higher anthocyanin accumulation in the *an3* and *yda* mutants is caused by lower or higher endogenous sugar accumulation, respectively, should be determined. The measurement of sugar metabolites in *an3*, Col-0, L*er, yda-1*, and *yda-2* seedlings was conducted using HPLC analysis. The results revealed that the concentrations of Suc and glucose, but not fructose, were significantly reduced in *an3* seedlings than in Col-0 seedlings (**Figures 6A–C**), whereas the concentrations of Suc, but not fructose and glucose, were higher in *yda* seedlings than in L*er* seedlings (**Figures 6E–G**). The anthocyanin biosynthetic pathways are strongly up-modulated along with Suc concentration, and the sugar-dependent upregulation of the anthocyanin synthesis pathway is Suc specific (Solfanelli et al., 2006; Jeong et al., 2010). Therefore, the lower Suc concentration in *an3* seedlings might specifically cause the lower anthocyanin accumulation, whereas the higher Suc concentration in *yda* seedlings might specifically cause the higher anthocyanin accumulation. Given that plant hormones, such as auxin and abscisic acid (Jeong et al., 2004; Hoth et al., 2010), cytokinin (Deikman and Hammer, 1995), gibberellins (Weiss et al., 1995) and ethylene (Morgan and Drew, 1997; Jeong et al., 2010), differentially regulate anthocyanin biosynthesis in whole plants and in cell suspensions (Ozeki and Komamine, 1986), whether *an3* and *yda* are the signaling/metabolism mutants of these plant hormones should be determined (Lukowitz et al., 2004). Therefore, the decreased or increased anthocyanin accumulation in the *an3* or *yda* mutants is mainly caused by the decreased or increased endogenous Suc concentration, respectively.

Exogenous Suc is applied in high concentrations to confirm this finding. That is, when grown on solid MS medium supplemented with 1% Suc, evident differences in anthocyanin accumulation can be observed on the epidermis of the abaxial side of leaves among the control, *an3* and *yda* seedlings (Supplementary Figure S1) (Meng, 2015). When grown on solid MS medium supplemented with 3% Suc, more evident differences can be detected on the epidermis of the corresponding organs among the control, *an3* and *yda* seedlings. That is, the epidermis of the abaxial side of the leaves of *an3-4* seedlings had lower anthocyanin accumulation than that of control seedlings, whereas the epidermis of the abaxial side of the leaves of *emb71* or *yda-1* seedlings had higher anthocyanin accumulation than that of control seedlings (**Figures 6Ia–d,K**). However, when grown on solid MS medium supplemented with 5% Suc, no evident differences in anthocyanin accumulation can be detected on the epidermis of the corresponding organs among the *an3*, wild-type and *yda* seedlings (Supplementary Figure S4), indicating that 5% Suc can restore abnormal anthocyanin accumulation in *an3* and *yda* mutants. Taken together, these results indicated that the decreased or increased anthocyanin accumulation in the *an3* or *yda* mutants is mainly caused by the decreased or increased endogenous Suc accumulation, respectively.

### The Decreased or Increased Endogenous Suc Accumulation in the *an3* or *yda* Mutants Are Due to the Increased or Decreased INV Activity, Respectively

Given that the entry of carbon from Suc into cellular metabolism in plants can potentially be catalyzed by INV (Lou et al., 2007), whether the decreased or increased endogenous Suc accumulation in the *an3* or *yda* mutant seedlings are caused by the increased or decreased INV activity, respectively, should be determined. Neutral INV is required for normal plant growth and development in *Arabidopsis* (Barratt et al., 2009), rice (Jia et al., 2008), and legumes (Welham et al., 2009). Neutral INV activity was assayed by ELISA. These findings indicated that the neutral INV activity in the *an3* and *yda* mutants was, on average, enhanced and reduced by approximately 30% and approximately 24%, respectively, compared with that in the controls (**Figures 6D,H**), implying that the lack of *AN3* and *YDA* activities might increase and decrease INV activity, respectively. Cell wall INV activity was determined to confirm this finding. NBT precipitate was observed in the elongation zone of *an3-4* roots, but not in the corresponding organs of wild-type roots (**Figure 6Ja,c**). By contrast, NBT precipitate was observed in the elongation zone of L*er* roots, but not in the corresponding organs of *yda-1* roots (**Figure 6Jb,d**). Controlled incubation of the *an3-4* mutant, wild-type, *yda-1* mutant and L*er* roots, which removed Suc, did not stain for INV (data not shown). The precipitate specifically indicates that cell wall INV has higher activity in the *an3-4* mutant than in the wild-type roots, whereas cell wall INV has lower activity in the *yda-1* mutant than in the L*er* roots. Taken together, these experimental results indicated that the lower endogenous Suc accumulation in the *an3* mutants was mainly caused by the enhanced INV activity, whereas the higher endogenous Suc accumulation in the *yda* mutants was mainly caused by the reduced INV activity.

### *AN3* Acts Genetically Upstream of *YDA* in Regulating Anthocyanin Accumulation

**FIGURE 7:**
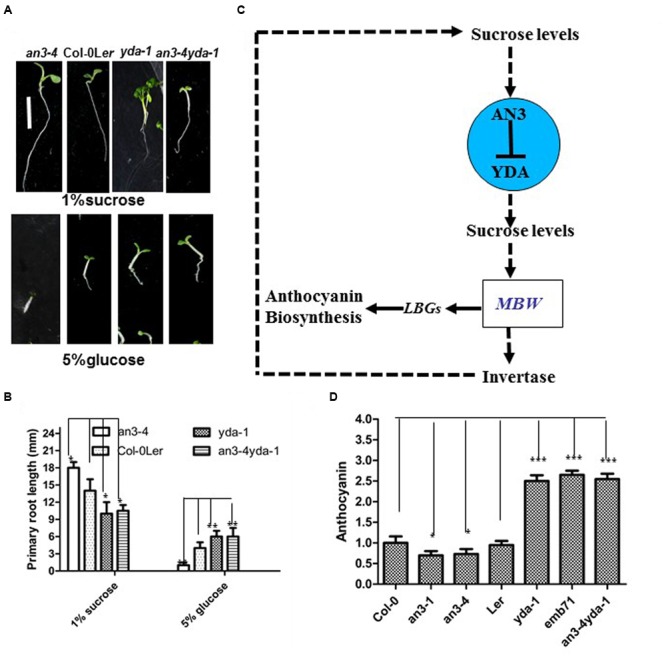
***EMB71/YDA* mutations differentially suppress *an3-4* mutant. (A)** Representative 5-day-old seedlings. Bar = 5 mm. **(B)** Bar graph exhibiting the difference in the root length. Error bars represent SD (*n* = 20). The above seedlings in **(A,B)** grown on solid MS medium with 1% sucrose and 5% glucose, and every kind of seedling have been indicated. Error bars represent SD (*n* = 17). Heteroscedastic *t*-test analysis showed significant differences (^∗^*P* < 0.05; ^∗∗^*P* < 0.01). Experiments were repeated two times with similar results (biological replicates). **(C)** A proposed model illustrating the regulation of anthocyanin accumulation by the *AN3-EMB71/YDA* gene cascade. The *AN3-EMB71/YDA* gene cascade tunes the sucrose levels, which may be as a result of the change of the invertase activity. The change of sucrose levels induces anthocyanin biosynthesis by unknown regulatory mechanisms. On the other hand, the MYB/bHLH/TTG1 (MBW) complex can directly regulate the invertase gene promoter, and in turn lead to the change of sucrose levels (Payyavula et al., 2013). This alternation may cause the change of stability in both AN3 and YDA in the *AN3-YDA* gene cascade. The solid lines indicate direct regulation, whereas the dotted lines indicate either indirect regulation or regulation in an unknown manner. **(D)** Bar graph exhibiting the difference in the anthocyanin accumulation between the every indicated seedlings grown under white light conditions on solid MS medium with 1% sucrose. Wild-type in normal is set as 1.0. Error bars represent SD (*n* = 13). Heteroscedastic *t*-test analysis showed significant differences (^∗^*P* < 0.05; ^∗∗∗^*P* < 0.001). Experiments were repeated two times with similar results (biological replicates).

The response of *an3-4* seedlings was hypersensitivity to high concentrations of glucose, whereas that of *yda-1* seedlings was less sensitivity to high concentrations of glucose (**Figures 7A,B**). The *an3-4* and *yda-1* mutants were less sensitive to high concentrations of glucose, which is comparable with the response of *yda-1* seedlings (**Figures 7A,B**). These findings indicated that *YDA* mutation inhibits the hypersensitivity of the *an3-4* mutant to high concentrations of glucose.

Double mutant analysis was performed to determine whether *AN3* acts genetically upstream of *YDA* in regulating anthocyanin accumulation. Specifically, *an3-4*, which presented a narrow rosette leaf (Horiguchi et al., 2005, 2011), was combined with *yda-1*, which showed a severely stunted phenotype compared with L*er* (Bergmann et al., 2004; Lukowitz et al., 2004). The results showed that *an3-4* and *yda-1* seedlings exhibited enhanced anthocyanin accumulation, similar to that of *yda-1* seedlings (**Figure 7D**). This crossing result indicated that *yda-1* is epistatic to *an3-4* in anthocyanin accumulation. These results also indicated that *YDA* mutation suppresses the reduced anthocyanin accumulation in *an3-4* mutant plants, implying that *AN3* acts genetically upstream of *YDA* in modulating the anthocyanin accumulation.

### *AN3-YDA* Plays Specific (but Generally Not Pleiotropic) Roles between Carbohydrate Synthesis/Status and Anthocyanin Biosynthesis

This study determined whether the expression levels of genes encoding flavonols and anthocyanins were changed in *an3* and *yda* mutants to verify the molecular basis of the changes of flavonol and anthocyanin levels. Microarray analysis (Horiguchi et al., 2011) revealed that the genes encoding a few regulators and flavonoid biosynthesis exhibited mostly two times lower expression levels in the *an3-4* mutant than in the wild-type seedlings (for example, F3′H, CHI, TRANSPARENT TESTA 19, flavonol synthase, UGT89C1 and UDP-GLUCOSYL TRANSFERASE 79B1) (Meng, 2015). Quantitative RT-PCR (Q-RT-PCR) analysis was performed to confirm the conclusion. The results indicated that the gene expression levels were evidently lower in the *an3-4* mutants than those in the wild-types (Supplementary Figure S6A), which is consistent with the results of microarray analysis (Horiguchi et al., 2011).

The regulatory and flavonoid biosynthetic genes that decreased in the *an3-4* mutant were investigated, and the results showed that these genes were mostly *EBGs* and *LBGs* (Meng, 2015). The expression levels of *EBGs* were unchanged in the *an3-4* mutant (Horiguchi et al., 2011). Therefore, whether AN3 protein is associated with the gene promoters of *LBGs* should be determined. First, this study determined whether these gene promoters contained TCTCTC or CACGTG motifs (Meng and Yao, 2015). The analysis indicated that the promoters of *F3′H* (*TT6*), *FLS* and *CHI*, but not *TT19, UGT79B1* and *UGT89B1*, contained CACGTG or TCTCTC motifs. Second, this study verified whether AN3 protein was associated with the promoters of *F3′H* (*TT6*), *FLS* and *CHI* by ChIP-PCR analysis. The findings indicated that AN3 protein was not associated with these gene promoters *in vivo* (Supplementary Figure S5).

Then, the microarray data of *YDA* were analyzed (Lukowitz et al., 2004), and the results revealed that, although the expression level of *CHI* was higher in the *emb71* mutants than in the control plants, the expression levels of *F3′H, FLS, TT19, UGT79B1*, and *UGT89C1* genes were not evidently different between *emb71* mutants and control plants. The findings of Q-RT-PCR analysis were consistent with those of microarray analysis (Supplementary Figure S6B) (Lukowitz et al., 2004). Given that the *yda-1* (or *emb71*) mutant exhibited overproduction of anthocyanin accumulation, why were the expression levels of these genes not evidently different between control and *yda-1* (or *emb71*) mutant plants? EMB71/YDA is the MEKK1/Ste11/Bck1 class of mitogen-activated protein kinase kinase kinases; thus, EMB71/YDA might regulate TT19, F3’H, UGT79B1, UGT89B1 and FLS at the post-translational level. Therefore, although the *yda-1* (or *emb71*) mutant exhibited overproduction of anthocyanin accumulation, the expression levels of these regulatory and flavonoid biosynthetic genes were not evidently different among wild-type and *yda-1* (or *emb71*) mutant seedlings. Taken together, these data indicated that the *AN3-YDA* gene cascade plays specific, but generally not pleiotropic, roles between carbohydrate synthesis/status and anthocyanin biosynthesis.

## Discussion

In this work, *an3* mutants exhibited lower anthocyanin accumulation than the wild-types, whereas *yda* mutants exhibited higher anthocyanin accumulation than the wild-types. The *an3* and *yda* mutants can disrupt the normal Suc levels as a result of higher or lower INV activity in *an3* and *yda* mutants, respectively. Thus, lower and higher anthocyanin accumulations were observed in *an3* and *yda* mutants, respectively. Taken together, the results showed that the *AN3-YDA* gene cascade may influence the INV activity, which affects the Suc levels. In turn, Suc levels can induce the change of anthocyanin levels to a certain extent via unknown regulatory mechanisms (**Figure 7C**). By contrast, the infiltration of the potato transcription factor anthocyanin 1 (StAN1) into tobacco leaves enhanced the gene expression of *SUSY* and *INV* (Payyavula et al., 2013). Thus, in *Arabidopsis*, certain factors of the MYB/bHLH/TTG1 (MBW) complex may affect the Suc levels by regulating the transcript levels of the INV gene (**Figure 7C**). Moreover, in the *AN3-YDA* gene cascade, the change of the Suc/glucose levels can cause the differential protein stability of AN3 and YDA, which again affects the MYB/bHLH/TTG1 (MBW) complex and anthocyanin levels of the *AN3-YDA* gene cascade regulating the Suc levels (**Figure 7C**).

Although the *AN3-YDA* gene cascade evidently affects the Suc levels, which probably triggers Suc signaling, its mechanism is still unknown. Jeong et al. (2010) reported that, although carbohydrate metabolism is not involved in the expression of the sugar transporter gene *SUC1*, their findings indicated that the redox status of photosynthetic electron transport represents a signal for anthocyanin induction. By contrast, *SUC1* is probably responsible for the enhanced Suc levels of the shoot for sugar sensing/signaling, but its specific role in enhancing shoot Suc levels remains elusive and requires further characterisation (Jeong et al., 2010). Moreover, *SUC1* plays a significant role in transporting exogenous Suc taken by proton-independent sugar transporters from the root tips (Chaudhuri et al., 2008). Jeong et al. (2010) reported that *SUC1* represents an integrator for signals provided by Suc. The identification of DELLA as a novel component in sucrose signaling, and the DELLA proteins act as a key positive regulator in the sucrose signaling pathway controlling anthocyanin biosynthesis (Li et al., 2014). Therefore, it is interesting whether the *AN3-YDA* gene cascade directly targets this sucrose signal integrator of SUC1 or the sucrose signaling component of DELLA for sucrose signal transduction. The interesting relationship awaits future research.

### The *AN3-YDA* Gene Cascade Presents Important Biological Functions in the Regulation of Anthocyanin Accumulation by Unknown Regulatory Mechanism

The *an3* and *yda* mutants exhibited lower and higher anthocyanin accumulations than the wild-types, respectively (**Figure 1**; Meng, 2015). Furthermore, the *an3* and *yda* double mutants exhibited higher anthocyanin accumulation than the wild-types (**Figure 7D**), indicating that *AN3* is upstream of *YDA* and that the *YDA* mutation significantly enhanced anthocyanin accumulation in the *an3-4* mutant. These findings indicated a negative relationship between *AN3* and *YDA* in the regulation of anthocyanin accumulation. Further analysis of the influence of AN3 associated with the *YDA* promoter *in vivo* (**Figures 2** and **3**) on glucose sensing (**Figure 4**) and the stability of AN3 and YDA protein on high concentrations of glucose (**Figures 5A,B**), on endogenous Suc content (**Figure 6**) and on INV activity (**Figure 6**) confirmed the negative regulatory effects of *AN3* on *YDA*.

Moreover, 5% sucrose can restore abnormal anthocyanin accumulation in *an3* and *yda* mutants (Supplementary Figure S4). Thus, the sugar signaling pathway is still functioning or, in case *an3* and *yda* would be involved in this signaling, there exists an alternative signaling pathway. However, these data do not support the conclusion that AN3 and YDA are part of the sucrose signaling. Thus, the AN3-YDA gene cascade obviously affect sugar levels, which will trigger the sugar signaling, the mechanism of which is still unknown.

Taken together, *AN3-YDA* gene cascade has important biological functions in the regulation of anthocyanin accumulation by unknown regulatory mechanism.

## Accession Numbers

Sequence data derived from this paper can be found in the *Arabidopsis* Genome Initiative database under the following accession numbers *AN3* (At5G28640) and *EMB71/YDA* (At1G63700).

## Author Contributions

L-SM designed experiments. L-SM performed the experiments. L-SM, Y-QL, and M-QL completed statistical analysis of data. L-SM and J-HJ wrote this manuscript.

## Conflict of Interest Statement

The authors declare that the research was conducted in the absence of any commercial or financial relationships that could be construed as a potential conflict of interest.
