# Corrigendum: The *Arabidopsis ANGUSTIFOLIA3-YODA* Gene Cascade Induces Anthocyanin Accumulation by Regulating Sucrose Levels

**DOI:** 10.3389/fpls.2017.01228

**Published:** 2017-07-10

**Authors:** Lai-Sheng Meng, Ying-Qiu Li, Meng-Qian Liu, Ji-Hong Jiang

**Affiliations:** ^1^The Key Laboratory of Biotechnology for Medicinal Plant of Jiangsu Province, School of Life Science, Jiangsu Normal UniversityXuzhou, China; ^2^Centre for Transformational Biotechnology of Medicinal and Food Plants, Jiangsu Normal University – Edinburgh UniversityXuzhou, China

**Keywords:** AN3/GIF1, *YODA (YDA)*, sucrose levels, anthocyanin accumulations, *Arabidopsis*

In the original article, there were mistakes in Figures 2F, 3J–L, 5C and 6I,J as published. The corrected Figures 2F, 3J–L, 5C and 6I,J appear below. The authors apologize for these errors and state that this does not change the scientific conclusions of the article in any way.

**Figure 2:**
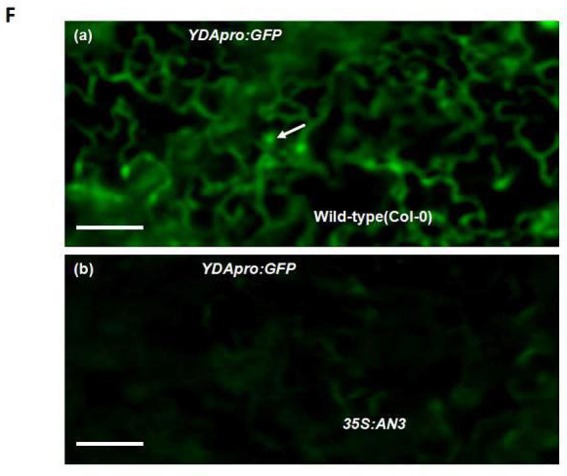
**(F)** *ProYDA:GFP* in WT(Col-0) **(a)** and *35S:AN3* **(b)** cotyledons, they are at same magnification. White arrows point to GFP-positive nuclei.

**Figure 3:**
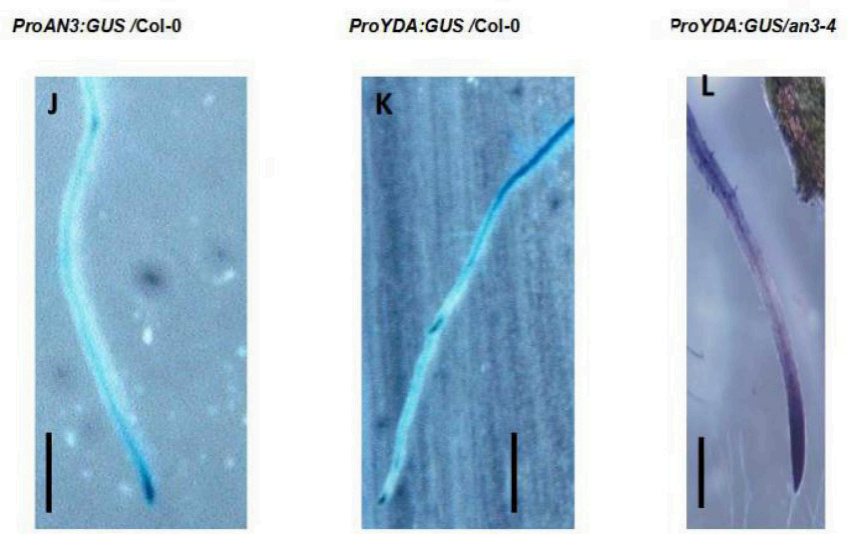
**(J–L)** Representative *ProAN3:GUS, ProEMB71/YDA:GUS*, and *ProEMB71/YDA:GUS* expression on primary roots in the wild-type background, in the wild-type background and in the *an3-4* background, respectively. Bar = 2.0 mm.

**Figure 5:**
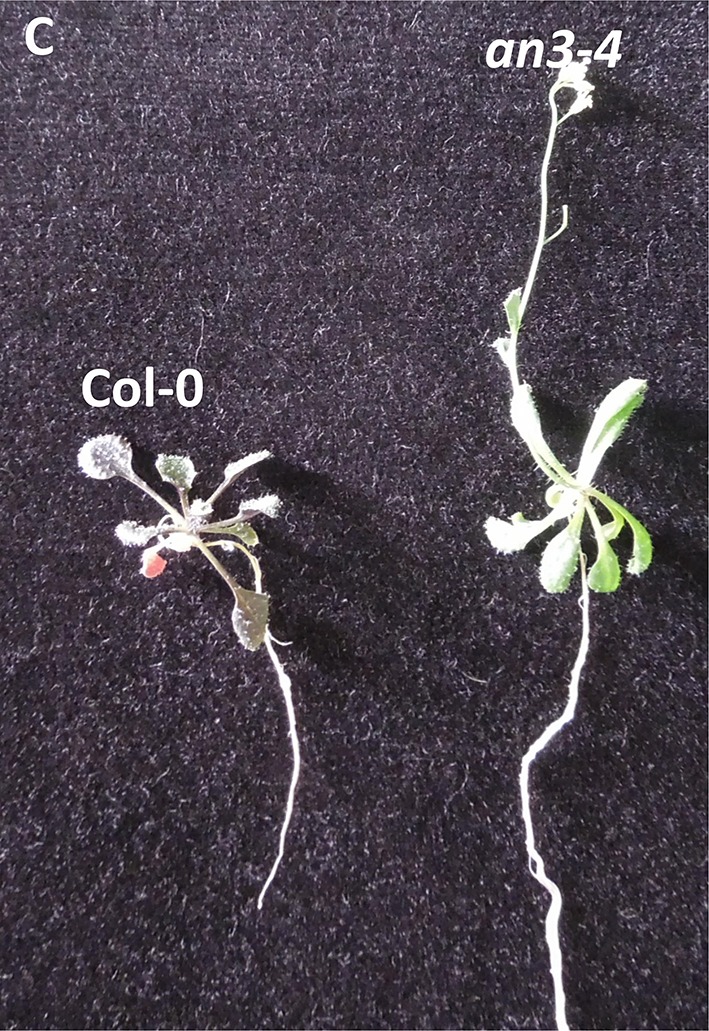
**(C)** Representative 5% glucose can restore the *an3-4* delayed flowering. Materials were grown on MS medium with 5% glucose for 4 weeks under long light (16 h light/8 h dark). Seedlings were from the same plate. Magnifications are the same.

**Figure 6:**
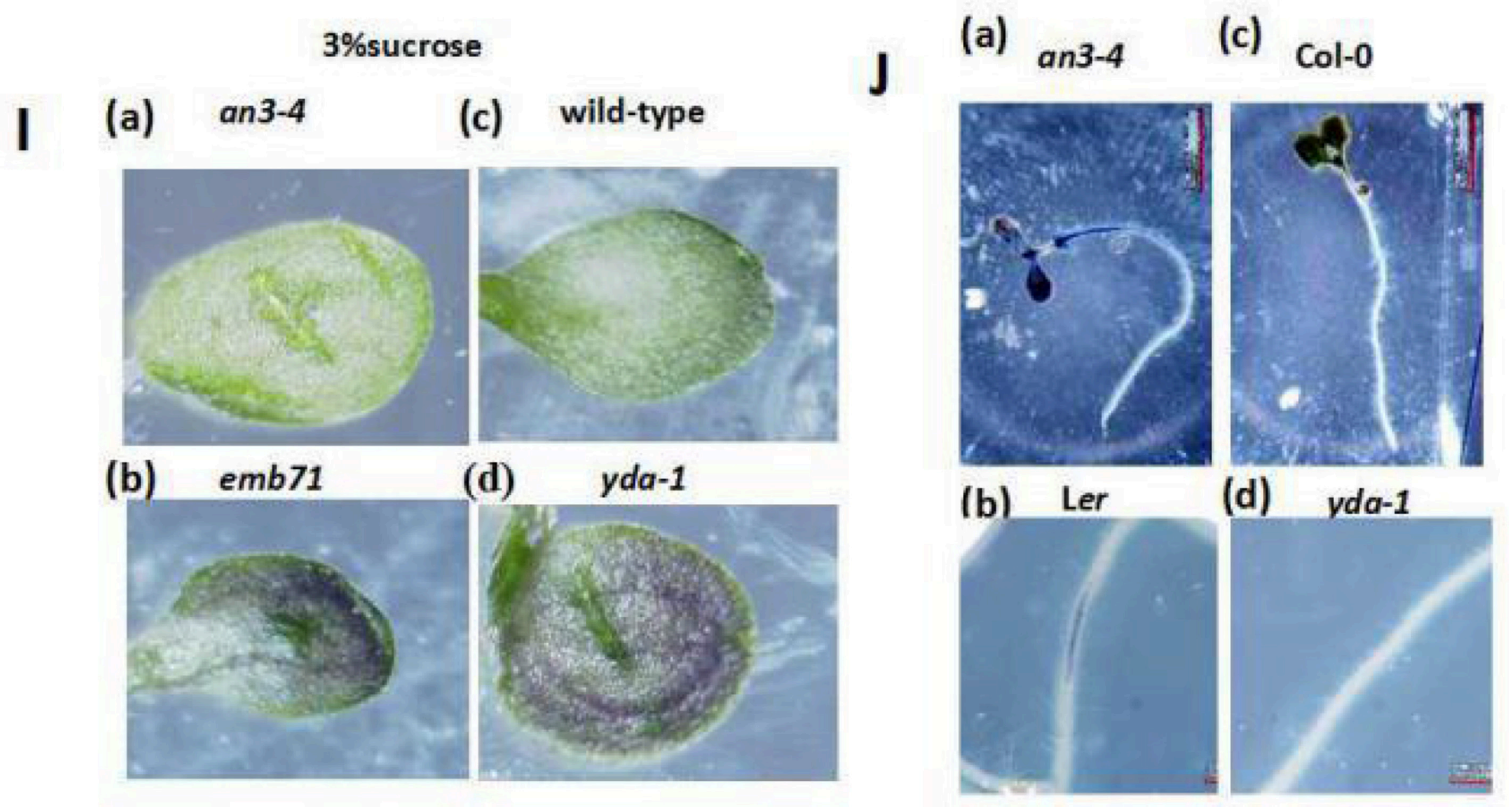
**(I)** Representative cotyledons of the 8-day-old *an3-4* **(a)**, *emb71* **(b)**, wild-type **(c)**, and *yda-1* **(d)** seedlings grown under long light (16L/8D) conditions on MS medium supplemented with 3% sucrose. **(J)** Representative nitroblue tetrazolium (NBT) precipitation in the 6-day-old *an3-4* **(a)**, L*er* **(b)**, Col-0 **(c)**, and *yda-1* **(d)** roots grown under long light (16 L/8 D) conditions on MS medium supplemented with 1% sucrose. Magnifications are the same.

## Conflict of interest statement

The authors declare that the research was conducted in the absence of any commercial or financial relationships that could be construed as a potential conflict of interest.

