# Supplementary material for: The Arabidopsis ANGUSTIFOLIA3-YODA Gene Cascade Induces Anthocyanin Accumulation by Regulating Sucrose Levels
Source: Front Plant Sci. 2016 Nov 22;7:1728. doi: 10.3389/fpls.2016.01728 (PMC5118565; doi:10.3389/fpls.2016.01728)
Supplement: Supplementary file 1 [file Image_1.PDF]

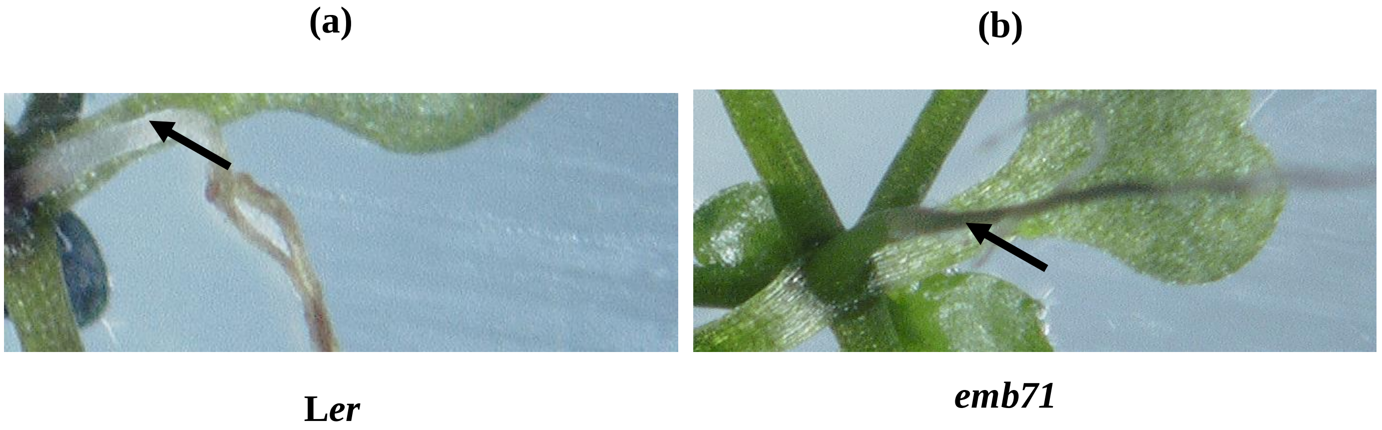

Supplemental Figure 1. L*er* and *emb71* Roots Had Different Anthocyanin Accumulation When Grown under White Light.
Representative 12-day-old L*er* (a) and *emb71*(b) primary roots grown under white light conditions on solid MS medium with 3% sucrose. Magnifications are the same.
Arrows indicate anthocyanin accumulation.

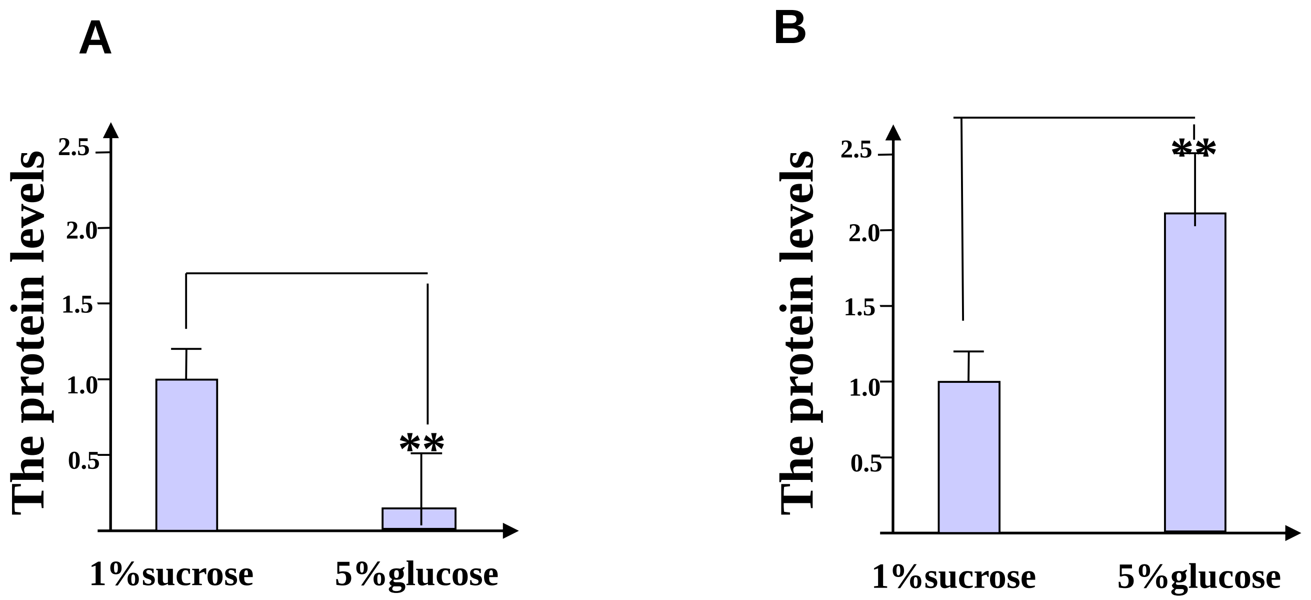

Supplemental figure 2. (A) and (B). The intensity of blotting was quantified through using Adobe Photoshop CS (Adobe Systems Inc.; San Jose, CA, USA) software, as has been described by Meng et al (2015b). Six blotting were measured. Data are means ± SD.The blotting intensity of the 1% sucrose of AN3-GFP (A) and 1% sucrose of YDA-GFP (B) was set as 1.0, respectively in (A and B).

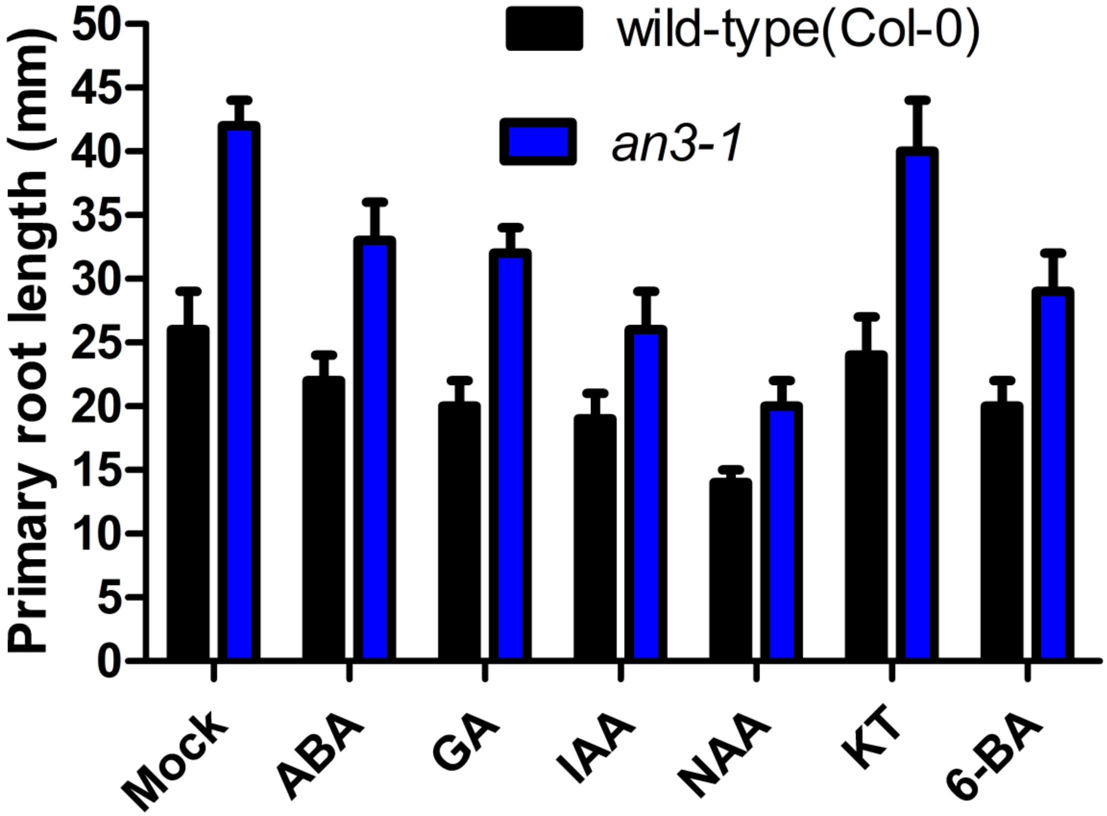

Supplemental Figure 3. Root Development in Wild-type (Col-0) and *an3-1* Seedlings is not Implicated in General Hormone Pathway.

Seeds were incubated on the solid MS medium supplemented without hormones for 5 days, and then they were transferred into the solid MS medium supplemented with the specified amount of hormones (ABA, abscisic acid; GA,:Gibberellin; IAA,Indole-3-acetic acid; NAA, 1-naphthlcetic acid; KT, 6-Furfurylaminopurine; 6-BA, 6-Benzylaminopurine). Total of approximate 40 roots of *an3-4* and Col-0 seedlings were measured . The values are average root lengths (mm) ± standard deviation. Mock indicates seedlings were transferred into the solid MS medium without hormones.

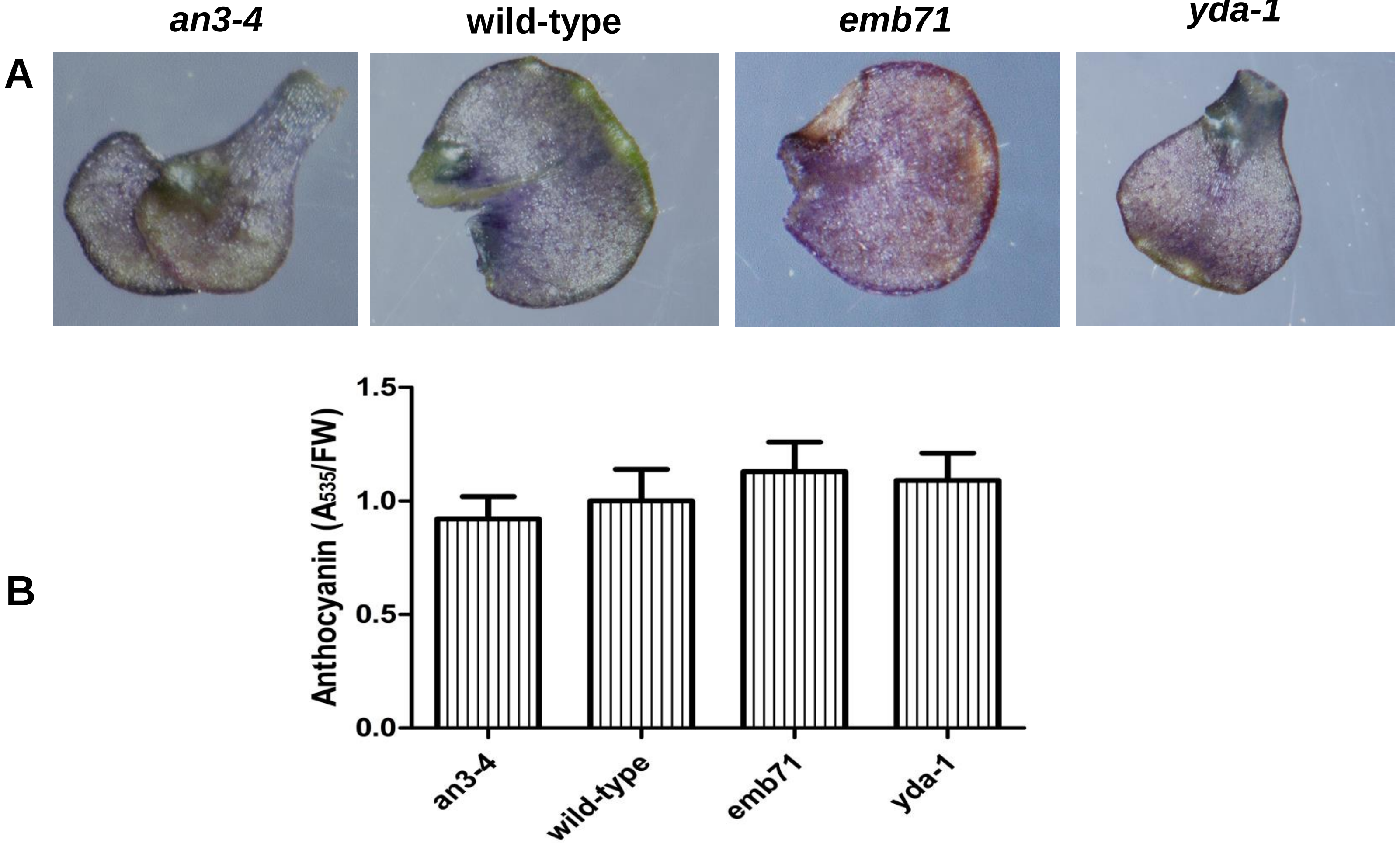

Supplemental Figure 4. 5% sucrose can restore abnormal anthocyanin accumulation in *an3* and *yda* mutants.

(A). Representative cotyledons of the 8-day-old *an3-4* (a), wild-type (b), *emb71* (c), and *yda-1* (d) seedlings grown under long light (16L/8D) conditions on MS medium supplemented with 5% sucrose.

(B). Bar graph exhibiting the difference in the anthocyanin accumulation between the *an3-4*, Wild-type (Col-0), *emb71* and *yda-1* cotyledons grown under white light conditions on MS medium supplemented with 5% sucrose.

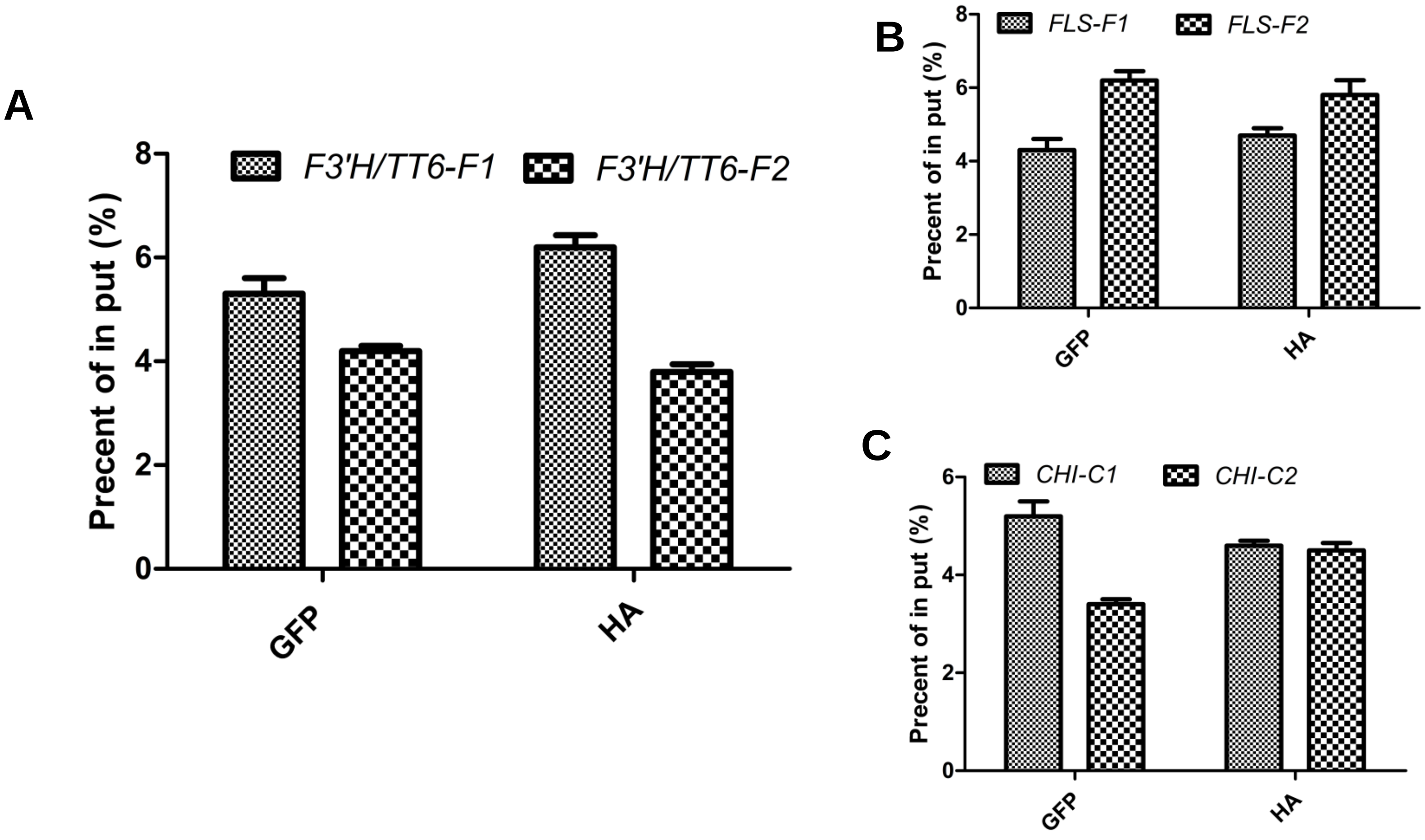

Supplemental Figure 5. AN3 is not associated to promoters of *TT6*, *FLS* and *CHI*.
(A)-(C). A chromatin immunoprecipitation (ChIP) analysis. Enrichment of particular chromatin regions with anti-HA antibody (as a control) or anti-GFP antibody in *35S:AN3-GFP* transgenic plants as detected by real-time PCR analysis. Quantifications were normalized to the expression of UBQ5. Error bars represent SD (n=3). In put is set as 100%.

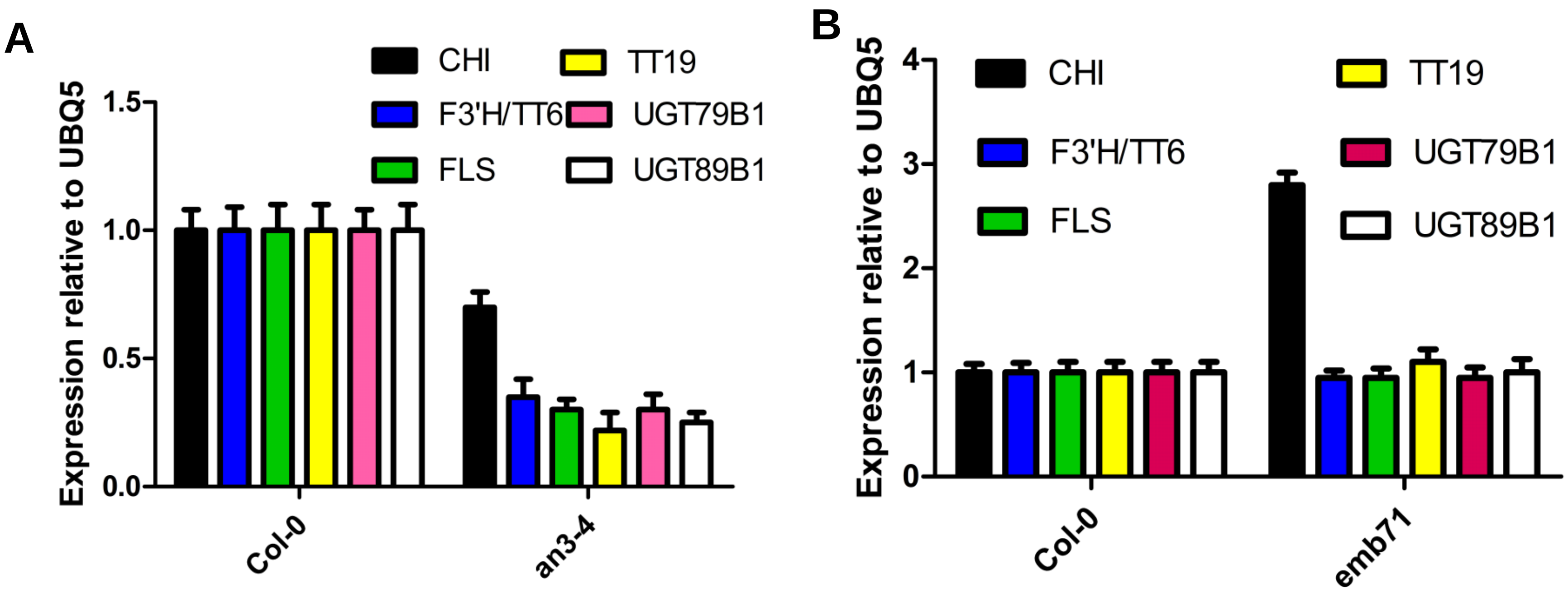

Supplemental Figure 6. Expression of Flavonoid Biosynthetic and Regulatory Genes. (A) and (B). Bar graph exhibiting the difference of expression of *CHI*, *F3'H (TT6)*, *FLS*, *TT19*, *UGT79B1* and *UGT89B1* between 20-day-old wild-type (Col-0) and *an3-4* seedlings (A); wild-type (Col-0) and *emb71* seedlings (B). Seedlings were grown under white light conditions. Data were from quantitative RT-PCR. Materials were from at least 5 independently propagated lines. And wild-type is set as 1.0. Quantifications were normalized to the expression of *UBQ5*. Error bars represent SD (n=3).
